# The “hot cross bun sign” in patients with autoimmune cerebellar ataxia: A case report and literature review

**DOI:** 10.3389/fneur.2022.979203

**Published:** 2022-08-19

**Authors:** Mange Liu, Haitao Ren, Nan Lin, Ying Tan, Siyuan Fan, Hongzhi Guan

**Affiliations:** ^1^Department of Neurology, Peking Union Medical College Hospital, Beijing, China; ^2^Chinese Academy of Medical Sciences, Peking Union Medical College, Beijing, China

**Keywords:** hot cross bun sign, anti-Ri antibody, paraneoplastic neurological syndrome (PNS), autoimmune cerebellar ataxia, case report

## Abstract

**Objectives:**

The “hot cross bun sign” (HCBs) on magnetic resonance imaging (MRI) has been initially considered specific for multiple system atrophy with cerebellar features. However, a number of other conditions have since been described, which may be associated with this imaging sign. We herein describe a patient with anti-Ri and paraneoplastic cerebellar ataxia, and review the association of the HCBs on imaging with various neurological autoimmune conditions.

**Methods:**

We report a 40-year-old woman with anti-Ri-associated paraneoplastic neurological syndrome and breast carcinoma, in whom brain MRI revealed the HCBs late in the disease course. We also reviewed similar cases reported in the literature.

**Results:**

The patient presented with cerebellar ataxia, polyneuropathy, and pyramidal signs. Although brain MRI was initially unremarkable, the HCBs and T2-weighted hyperintensity of the bilateral middle cerebellar peduncles were observed at later follow-up. Anti-Ri was detected in the serum and cerebrospinal fluid. Breast adenocarcinoma was confirmed *via* an axillary lymph node biopsy. Her symptoms partially resolved after the first corticosteroid pulse. However, subsequent immunotherapy and tumor treatments were ineffective. Four autoimmune cerebellar ataxia cases with the HCBs (two paraneoplastic and two non-paraneoplastic) were identified in the literature.

**Discussion:**

The HCBs can be associated with paraneoplastic and non-paraneoplastic cerebellar ataxia, which may reflect neurodegeneration secondary to autoimmune injury. Thus, the HCBs should not be considered a contraindication for autoimmune cerebellar syndrome.

## Introduction

The “hot cross bun sign” (HCBs) refers to a characteristic cruciform pontine T2-weighted hyperintensity evident on brain magnetic resonance imaging (MRI) and is suggestive of multiple system atrophy with cerebellar features (MSA-C). MSA-C is a neurodegenerative alpha-synucleinopathy and a common cause of adult-onset sporadic cerebellar ataxia (1). The diagnostic specificity of the HCBs and middle cerebellar peduncular hyperintensity on MRI for MSA-C is as high as 98.5% (2). However, the HCBs has also been reported in patients with neurological autoimmunity. To help improve the differential diagnosis spectrum of the HCBs, here, we present a patient with anti-Ri-related paraneoplastic neurological syndrome (PNS) and breast cancer who showed the HCBs. We also provide a review of patients with the HCBs associated with autoimmune etiologies.

## Case presentation

**Figure 1:**
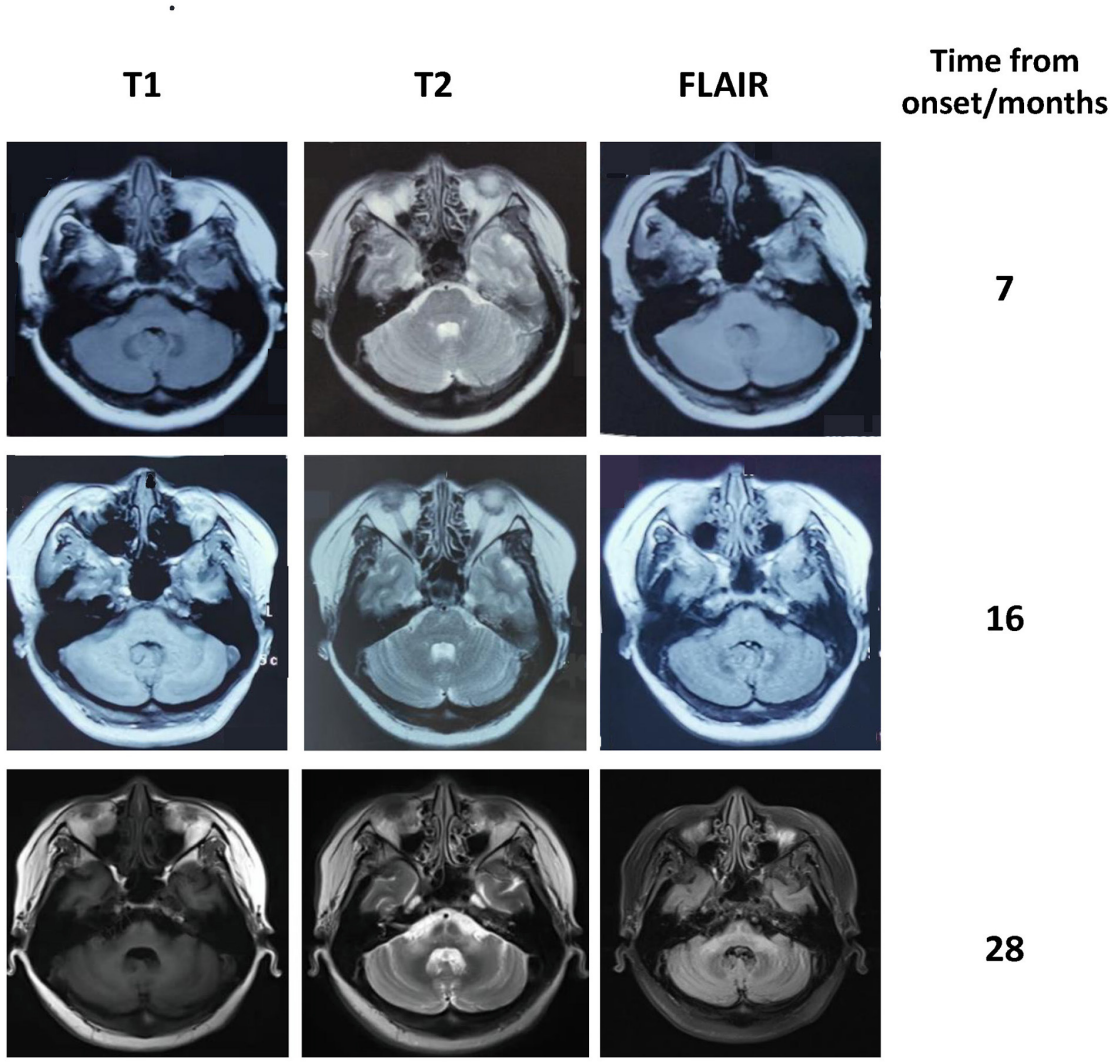
Brain MRI of our patient showing progressive atrophy of the cerebellum and the middle cerebellar peduncle. The hot cross bun sign and the abnormal signal in the middle cerebellar peduncles became more pronounced over time.

A 40-year-old woman presented with paresthesia and weakness in all four limbs for 2 years and an unstable gait for 1 year. Electromyography conducted 7 months after the disease onset showed neurogenic change, while brain MRI performed was unremarkable. Ganglioside antibodies in the serum and cerebrospinal fluid (CSF) were both negative. Empirical corticosteroids significantly improved her symptoms. However, the numbness and quadriplegia reappeared during corticosteroid weaning 4 months later, and she gradually developed dizziness, an unsteady gait, and diplopia. Brain MRI performed at 16 months after the disease onset revealed T2 hyperintensity in the middle of the pons (Figure 1). There was no improvement with pulse glucocorticoid therapy and intravenous immunoglobulin, and she developed slurred speech and dysphagia.

Physical examination revealed dysarthria, paresis of left eye adduction, diplopia, and nystagmus. The lower extremities exhibited weakness, hyporeflexia, and Babinski's signs. The finger-to-nose and knee-heel-shin tests revealed slowness and intentional tremors. She could not walk independently and was wheelchair-bound. Her sensation was intact. The Scale for the Assessment and Rating of Ataxia score was 33.5, and the modified Rankin Scale score was 4.

Complete blood count, biochemical tests, and screening for infection, toxins, and metabolic and systemic autoimmune diseases were unremarkable. There was an increased CSF white blood cell count (22 cells/μL) and positive oligoclonal bands. PNS autoantibody assays (for the Hu/Yo/Ri/Ma2/Ta/CV2/Tr/Zic4/SOX1/amphiphysin antibodies) revealed anti-Ri in the serum and CSF. Electromyography demonstrated sensory polyneuropathy. Spinal MRI was unremarkable. Hypermetabolism and enlargement of the left axillary lymph nodes were apparent on positron emission tomography-computed tomography. Biopsies revealed breast adenocarcinoma metastases [CK7 (+), ER (++), PR (–), HER-2 (+)]. No hypermetabolism in positron emission tomography-computed tomography was observed in the mammary glands, and pathological investigation after left mammectomy revealed no malignancy. 18F-fluorodeoxyglucose uptake of the cerebellum was decreased.

**Figure 2:**
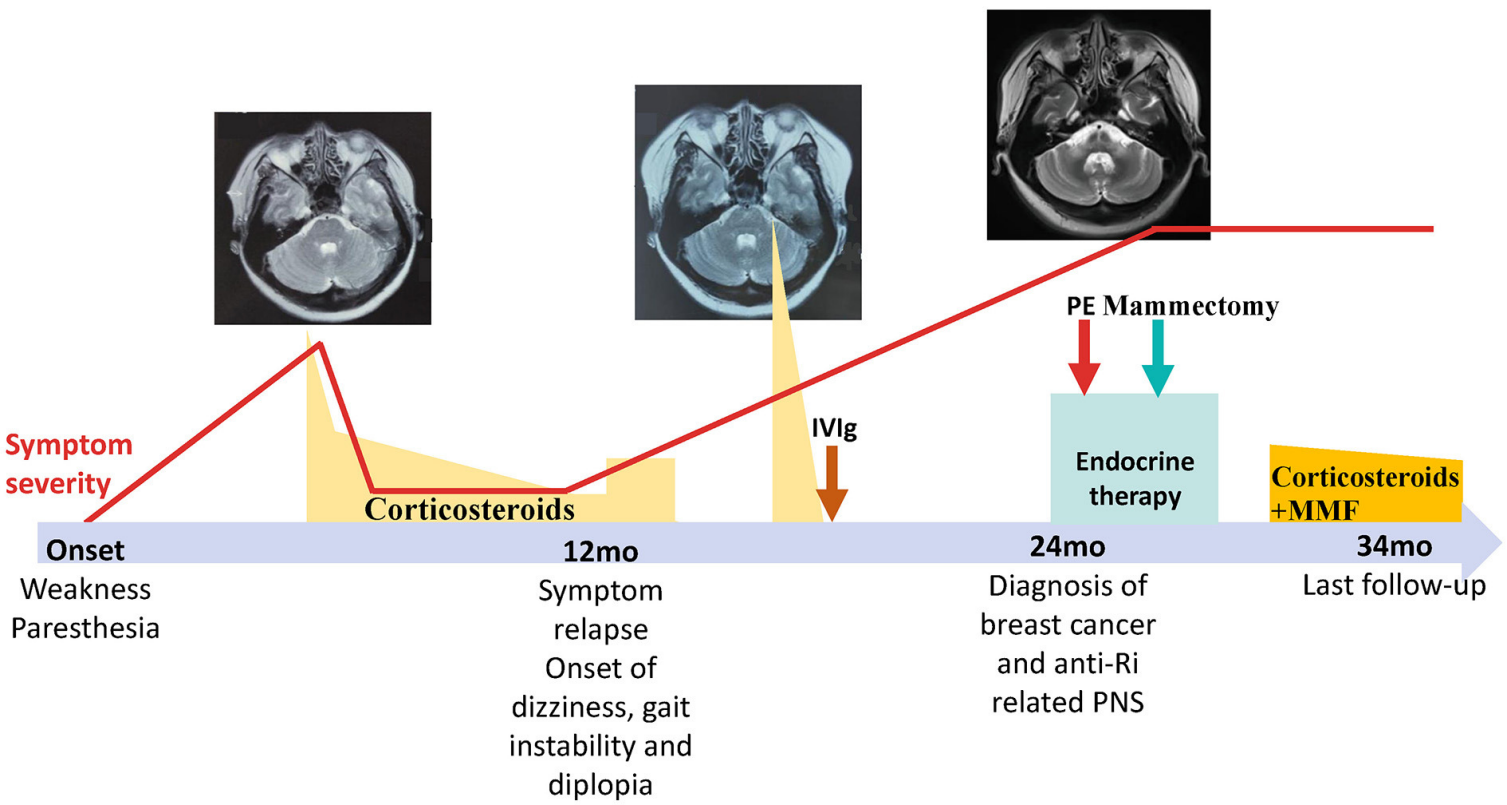
The timeline of the present case.

The patient was diagnosed with PNS with breast carcinoma. Endocrine therapy with goserelin and letrozole and plasma exchange were performed. However, her neurological symptoms worsened. Follow-up brain MRI revealed the HCBs, with signal change in the bilateral middle cerebellar peduncles and widened cerebellar sulci (Figure 1). Her symptom was stabilized by a repeated course of corticosteroid and mycophenolate mofetil treatment, after which CSF pleocytosis improved (white blood cell count, 2 cells/ul). However, CSF oligoclonal bands and anti-Ri in the serum and CSF remained positive. Her modified Rankin Scale score evaluated 34 months after the disease onset was 4. The clinical course of the patient is summarized in Figure 2.

## Discussion

Herein, we report a patient with paraneoplastic cerebellar ataxia and polyneuropathy in whom brain MRI showed the HCBs and bilateral middle cerebellar peduncle hyperintensities, imitating MSA-C. To our knowledge, this is the first report of the HCBs in a patient with PNS related to anti-Ri antibodies.

As the targets of anti-Ri are intracellular RNA-binding proteins encoded by Nova-1 and Nova-2 genes, direct pathogenicity of anti-Ri antibodies is unlikely, and T lymphocyte-mediated neuronal damage is considered the main pathogenic mechanism (3). In pathological studies, lymphocytic infiltration was detected in the cerebellum, brainstem, and neocortex, with prominent Purkinje cell loss (4). Most patients with anti-Ri antibodies have unremarkable brain MRI findings, although some abnormalities have been reported, including signal changes in the brainstem, the medial temporal and insular lobes, and reversible lesions in the pontine tegmentum (3, 5). This seems in line with the multifocal neurological abnormalities involving cerebellar ataxia, opsoclonus-myoclonus syndrome, jaw dystonia, and Parkinsonism (3, 6, 7). As for the present case, peripheral neuropathy was frequently reported, although Ri is exclusively expressed in the central nervous system (3).

Historically, the HCBs was considered a marker of MSA-C (8). However, its diagnostic specificity is now being questioned because of its identification in a range of other diseases. The pathogenic conditions underlying the HCBs include infectious (e.g., progressive multifocal leukoencephalopathy, Creutzfeldt-Jakob disease) (9–11), hereditary (e.g., spinocerebellar ataxia type 1, spinocerebellar ataxia type 3, cerebrotendinous xanthomatosis, oculodentodigital dysplasia, fragile X tremor ataxia syndrome) (12–16) or inflammatory (17) disorders. The HCBs secondary to leptomeningeal metastasis and infarction was also reported (18–20). The sign is thought to reflect Wallerian degeneration of the transverse pontocerebellar fibers and neuronal loss in the pontine raphe, with preservation of the pontine tegmentum, superior ventral cerebellar peduncles, and the bilateral corticospinal tracts (19, 21, 22). Gliosis of the reticular formation in the middle of the pontine and the pontocerebellar fiber also contributes to the HCBs (23).

In our systematic review, we searched the PubMed and EMBASE databases using “hot cross bun sign” OR “cruciform” OR “cruciate” and “autoimmune” OR “encephalitis” OR “rhombencephalitis” OR “paraneoplastic” as keywords. We excluded patients with central nervous system inflammatory demyelinating diseases because of their distinct pathogenicity. Four autoimmune cerebellar ataxia patients with the HCBs were identified in the literature (Table 1). Two of the patients were paraneoplastic, and two were non-paraneoplastic with anti-Homer 3 antibodies (24–26). All four patients presented with cerebellar ataxia, with or without other neurological abnormalities, including diplopia, pyramidal sign, rapid eye movement sleep behavior disorder, and sensorineural hearing loss. Notably, the HCBs usually appeared later in the disease course, with or without middle cerebellar peduncle lesions, while brain MRI at presentation was typically unremarkable or showed cerebellar atrophy. The patients generally had a poor outcome despite a wide range of immunological and oncological treatments.

**Table 1:** A summary of patients with autoimmune cerebellar ataxia showing the HCBs.

**Patient (sex/age at onset)**	**Onset**	**Neurological syndrome**	**Neuronal antibody/Malignancy**	**MRI at presentation/ at last follow-up**	**CSF WBC (/μL)/protein (g/L)/OCB**	**Treatment (outcome)**	**mRS/ SARA at last follow-up (mo from onset)**
1 (F/38, this study)	Subacute	Cerebellar ataxia, diplopia, pyramidal sign, polyneuropathy	Anti-Ri/Breast cancer	Unremarkable/ Cerebellar atrophy, HCBs, T2-hyperintensity in MCPs	22/0.45/+	CS (improved initially but deteriorated later), IVIg, PLEX and endocrine therapy (deteriorated)	4/33.5 (28)
2 (51/F(26))	Subacute	Cerebellar ataxia	Anti-amphiphysin/ Breast cancer	HCBs, T2-hyperintensity in MCPs/ extension of MCP lesion to the midbrain	NA	NA	NA
3 (M/42(25))	Subacute	Cerebellar ataxia, sensorineural hearing loss	Anti-KLHL-11/ Seminoma	Cerebellar atrophy/ Cerebellar and brainstem atrophy, HCBs, T2-hyperintensity in MCPs, hypointensity on SWI in the substantia nigra, red nucleus and dentate nuclei	8/0.52/+	Tumor resection and chemotherapy (stabilization), CS and IVIg (stabilization)	4/21 (96)
4 (F/50(24))	Subacute	Cerebellar ataxia, RBD	Anti-Homer 3/None	Unremarkable/ Cerebellum and pons atrophy, HCBs	2/0.3/-	CS, MMF (partial recovery)	2/12(31)
5 (M/65(24))	Insidious	Cerebellar ataxia, RBD	Anti-Homer 3/None	Cerebellum and pons atrophy/ Cerebellum, pons and cerebellum peduncle atrophy, HCBs	30/1.136/- ^*^	IVIg, CS, PLEX (deteriorated)	4/NA (64)

CS, corticosteroid; CSF, cerebrospinal fluid; HCBs, hot cross bun sign; IVIg, intravenous immunoglobulin; KLHL-11, kelch-like protein 11; MCP, middle cerebellar peduncle; MMF, mycophenolate mofetil; mo, month; MRI, magnetic resonance imaging; mRS, modified Rankin Scale; NA, not available; OCB, oligoclonal bands; PLEX, plasma exchange; SARA, Scale for the assessment and rating of ataxia; SWI, susceptibility-weighted imaging.

* Results affected by traumatic lumbar puncture.

In patients with PNS, the HCBs was first reported in a patient with kelch-like protein 11 (KLHL11) antibody (25). Interestingly, that patient and the present patient both presented with occult malignancies and lymph node metastases. Thus, a propensity toward lymphatic metastasis may facilitate the presentation of tumor autoantigens to the immune system, triggering cross reaction with the nervous tissue. The strong immune response may also promote regression of the primary tumor (27).

The patients with Homer 3 antibodies could show rapid eye movement sleep behavior disorder and cerebellar ataxia, in addition to the HCBs. Thus, it can be difficult to differentiate this disorder from degenerative MSA-C, particularly when the onset is insidious or an inflammatory CSF profile is absent (24, 28). Antineuronal autoantibody testing is important for identifying such potentially treatable etiologies.

The HCBs usually appears late in the disease course, and patients generally experience poor outcomes despite immunological and oncological treatments. In our patient, immunotherapy and tumor treatment failed to improve the cerebellar syndrome. Nevertheless, stabilization of the ataxia and alleviation of CSF inflammation support an immune-mediated etiology and treatment efficacy. In these circumstances, irreversible neuronal loss secondary to autoimmune destruction of the cerebellum and related structures may have already occurred, as suggested by the prominent cerebellar atrophy and the HCBs on brain MRI. However, there is some evidence that the HCBs can be reversed after immunotherapy, such as in neuromyelitis optica spectrum disorders (1).

## Conclusions

This present case provides further evidence that the HCBs is not specific for MSA-C but, rather, can also appear in paraneoplastic or non-paraneoplastic autoimmune cerebellar ataxia, including PNS with anti-Ri. When this imaging characteristic is associated with the subacute/acute disease onset, an inflammatory CSF profile, and the absence of autonomic dysfunction (which were atypical for MSA-C), clinicians should consider the potential for autoimmune and paraneoplastic etiologies, and CSF examinations, neuronal autoantibody assays, and malignancy screening should be considered.

## Data availability statement

The raw data supporting the conclusions of this article will be made available by the authors, without undue reservation.

## Ethics statement

This study was approved by the Ethics Committee of Peking Union Medical College Hospital (JS-891). The patients/participants provided their written informed consent to participate in this study. Written informed consent was obtained from the individual(s) for the publication of any potentially identifiable images or data included in this article.

## Author contributions

ML and HR drafted the manuscript for intellectual content and collected and analyzed the data. NL and YT collected and analyzed the data and revised the manuscript for intellectual content. SF and HG revised the manuscript for intellectual content. All authors contributed to the article and approved the submitted version.

## Funding

This study is funded by CAMS Innovation Fund for Medical Sciences (CIFMS #2021-I2M-1-003).

## Conflict of interest

The authors declare that the research was conducted in the absence of any commercial or financial relationships that could be construed as a potential conflict of interest.

## Publisher's note

All claims expressed in this article are solely those of the authors and do not necessarily represent those of their affiliated organizations, or those of the publisher, the editors and the reviewers. Any product that may be evaluated in this article, or claim that may be made by its manufacturer, is not guaranteed or endorsed by the publisher.

## Abbreviations

CSF: cerebrospinal fluid
HCBs: hot cross bun sign
MRI: magnetic resonance imaging
MSA-C: multiple system atrophy with cerebellar features
PNS: paraneoplastic neurological syndrome.
